# Morphologic Features and Clinical Outcomes of Acinar Cell Carcinoma of the Pancreas: A Multicenter Retrospective Study of 37 Patients in South Korea †

**DOI:** 10.3390/curroncol33060367

**Published:** 2026-06-18

**Authors:** Yoon Suk Lee, Woo Hyun Paik, Min Kyu Jung, Jung Won Chun, Young Hoon Choi, Joo Kyung Park, Kyu Hyun Paik, In Seok Lee, Sang Myung Woo, Jin-Hyeok Hwang

**Affiliations:** 1 Department of Internal Medicine, Ilsan Paik Hospital, Inje University College of Medicine, Goyang 10380, Republic of Korea; lys0326@snu.ac.kr; 2 Department of Internal Medicine, Seoul Metropolitan Government-Seoul National University Boramae Medical Center, Seoul 07061, Republic of Korea; 3 Department of Internal Medicine and Liver Research Institute, Seoul National University Hospital, Seoul 03080, Republic of Korea; iatrus@snu.ac.kr; 4 Department of Internal Medicine, Kyungpook National University Hospital, Daegu 41944, Republic of Korea; minky1973@knu.ac.kr; 5 Research Institute, Center for Liver and Pancreatobiliary Cancer, National Cancer Center, 323 Ilsan-ro, Ilsandong-gu, Goyang 10408, Republic of Korea; 6 Department of Medicine, Samsung Medical Center, Sungkyunkwan University School of Medicine, Seoul 06351, Republic of Korea; younghoon.choi@samsung.com (Y.H.C.); jkpark119@skku.edu (J.K.P.); 7 Department of Internal Medicine, College of Medicine, The Catholic University of Korea, Seoul 06591, Republic of Korea; qhyun515@naver.com (K.H.P.); isle@catholic.ac.kr (I.S.L.); 8 Department of Internal Medicine, Seoul National University Bundang Hospital, Seoul National University College of Medicine, 82, Gumi-ro 173 Beon-gil, Bundang-gu, Seongnam 13620, Republic of Korea

**Keywords:** acinar cell carcinoma, pancreatic neoplasms, Republic of Korea, Multicenter Studies as Topic

## Abstract

**Background:** The clinical characteristics of pancreatic acinar cell carcinoma (ACC) remain poorly defined due to its rarity. This study aimed to evaluate the morphological features and clinical outcomes of pancreatic ACC. **Method:** This multicenter retrospective study analyzed clinical data from seven referral hospitals. Electronic medical records were comprehensively reviewed to extract patient data. Survival outcomes were calculated from the date of pathologic confirmation of ACC. **Results:** Of the 37 patients, 28 (75.7%) were male. The age distribution at diagnosis ranged widely from 12 to 86 years, with a median of 62.0 years; seven patients (18.9%) were aged under 50 years. Morphologically, 24 patients (64.9%) presented with solid masses, whereas four had cystic masses and four exhibited mixed solid and cystic components. Regarding tumor resectability, 19 patients (51.4%) had resectable disease, 7 (18.9%) were locally advanced, and 11 (29.7%) were metastatic. In terms of treatment, 22 patients (59.4%) underwent surgical resection, 12 (32.4%) received palliative chemotherapy, and the remainder received best supportive care. In the surgical resection group, the median OS was not reached, demonstrating significantly prolonged survival (mean OS, 7.6 years; 5-year OS rate, 51%). In contrast, the median OS was 0.9 years in the palliative chemotherapy group and 0.1 years in the best supportive care group (*p* = 0.040). **Conclusions:** Pancreatic ACC showed a broad age distribution, with approximately 20% of patients aged <50 years, and pleomorphic morphological features, including solid, cystic, and mixed patterns. Patients who underwent surgical resection demonstrated favorable long-term survival outcomes compared to historical data for pancreatic ductal adenocarcinoma.

## Simple Summary

Pancreatic acinar cell carcinoma (ACC) is an exceedingly rare malignancy; therefore, its morphologic features and clinical outcomes of pancreatic ACC were poorly defined. This multicenter retrospective study elucidates several crucial findings for clinical practice. Demographically, pancreatic ACC exhibits a broader age distribution, with approximately 20% of our cohort diagnosed under the age of 50. Radiologically, these tumors characteristically present as large, hypo-enhancing solid masses; however, a subset of pancreatic ACCs may exhibit an arterial hyperenhancement pattern or pleomorphic morphologies encompassing solid, cystic or mixed components. More importantly, despite their large size, pancreatic ACCs are relatively well-circumscribed and have a high resectability rate. Consequently, surgical resection may afford a potential for long-term survival.

## 1. Introduction

Pancreatic neoplasms are classified into ductal, neuroendocrine, and acinar lineages based on their cellular origin. Among these, acinar cell carcinoma (ACC) of the pancreas, which arises from enzyme-producing acinar cells, is a rare malignancy, accounting for only 1% to 2% of all exocrine pancreatic tumors. While the clinical behavior of pancreatic ACC remains poorly defined, pancreatic ductal adenocarcinoma (PDAC), originating from pancreatic ductal epithelium, is the most prevalent malignancy, representing approximately 85% to 90% of all pancreatic neoplasms. PDAC typically affects patients older than 60 years and is rarely observed in individuals younger than 50 years [1]. In contrast, pancreatic ACC exhibits a broader age distribution with bimodal peaks in childhood and late adulthood; approximately 15% of cases occur in pediatric neoplasms [2,3].

Regarding clinical presentation, obstructive jaundice or cholangitis is less frequently associated with pancreatic ACC than with PDAC. However, pancreatic ACC can induce a pancreatic panniculitis related to the high levels of serum lipase [4]. Although the survival outcomes for pancreatic ACC appear more favorable than those for PDAC, they are generally inferior to those for pancreatic neuroendocrine tumors [1,2,5]. Nevertheless, these prognostic characteristics require further elucidation, as current evidence is largely anecdotal due to the rarity of pancreatic ACC. Furthermore, while PDAC typically presents as an ill-defined solid mass with biliary or pancreatic duct invasion—leading to obstructive jaundice or upstreaming p-duct dilation—the morphological features and invasive patterns of pancreatic ACC remain poorly characterized. Therefore, this study aimed to investigate the morphologic characteristics and clinical outcomes of pancreatic ACCs by analyzing long-term survival in relation to surgical or non-surgical treatments.

## 2. Methods

### 2.1. Study Design and Patient Selection

**Figure 1:**
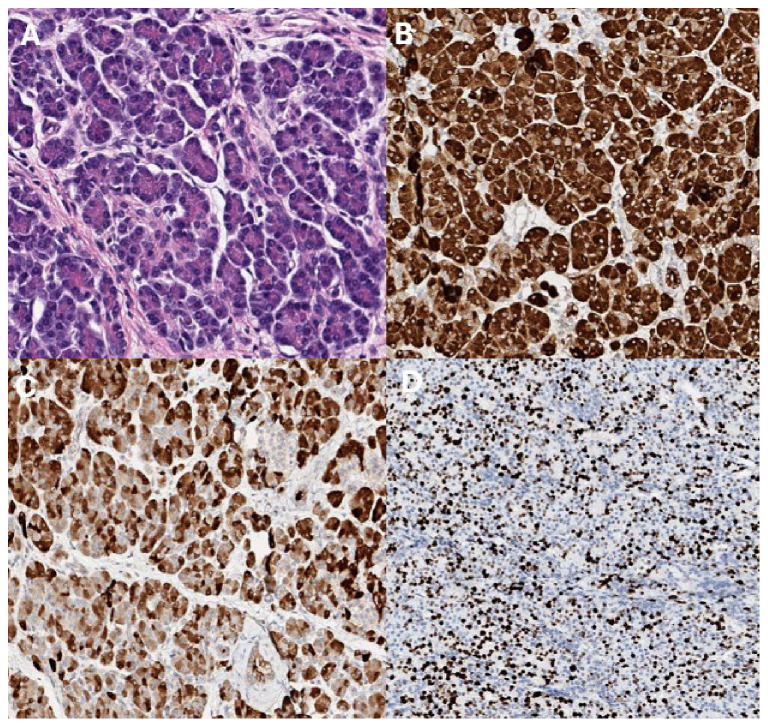
Representative histologic and immunohistochemical features of pancreatic acinar cell carcinoma. (**A**) Hematoxylin and eosin staining (×400) shows solid/acinar architecture with tumor cells containing granular eosinophilic cytoplasm. (**B**) Immunohistochemistry for BCL10 (×400) shows diffuse cytoplasmic positivity for the acinar marker BCL10. (**C**) Immunohistochemistry for chymotrypsin C (×400) shows strong cytoplasmic positivity. (**D**) Immunohistochemistry for Ki-67 (×200) shows a significantly increased proliferative index in tumor cells, approximately 50%.

This was a multicenter, retrospective study conducted through a comprehensive review of electronic medical records of patients diagnosed with pancreatic ACC between January 2010 and December 2023. To ensure diagnostic accuracy, the inclusion criteria were strictly limited to patients with histologically confirmed pancreatic ACC, showing BLC10 positivity in immunostaining. Representative histopathological images are presented in Figure 1. Diagnostic confirmation was established via surgical resection or biopsy. A total of 37 patients were identified across seven major academic referral hospitals in South Korea: National Cancer Center (NCC), Seoul National University (SNU), Seoul National University Bundang Hospital (SNUBH), Samsung Medical Center (SMC), The Catholic University of Korea St. Mary’s Hospital (CMC), and Kyungpook National University Hospital (KNUH) and Ilsan Paik Hospital (ISPAIK). The study protocol was approved by the Institutional Review Board, and informed consent was waived due to the retrospective design (IRB number NCC: NCC 2023-0038 approved at 14 February 2023; SNUH: 2308-077-1458 approved at 16 August 2023, SNUBH: B-2308-844-106 approved at 27 July 2023; SMC: SMC 2023-06-053-001 approved at 11 August 2023; CMC: KC22ONDI0067 approved at 4 February 2022; KNUH: KNUH 2023-06-003 approved at 26 June 2023; ISPAIK: ISPAIK2023-06-010 approved at 6 March 2023).

### 2.2. Data Collection and Survival Analysis

Baseline characteristics, including patient demographics (age and sex), clinical symptoms at presentation, and initial laboratory findings, were extracted for all patients. To evaluate the morphological features of the pancreatic ACC, tumor characteristics including tumor texture, internal composition, and relationship with surrounding structures were primarily analyzed using computed tomography (CT) images. Treatment strategies, such as surgical intervention and systemic chemotherapy, were reviewed to assess their impact on survival outcomes. The metabolic activity of tumors was quantitatively evaluated using the maximum standardized uptake value (SUVmax) from 18F-FDG PET-CT scans. The threshold of SUVmax ≥ 5.0 was primarily defined as ‘high-uptake.’ However, given the multi-center nature of this study, minor institutional adjustments to this cut-off value of the ‘high-uptake’ were permitted to account for variations in equipment specifications and diagnostic protocols across participating centers. Survival outcomes were assessed by respectability and treatment strategies to determine the prognosis of pancreatic ACCs. Overall survival (OS) time was defined as the interval from the date of pathologic diagnosis to the date of death from any cause or the last follow-up visit.

All statistical analyses were performed using SPSS software, version 20.0 (IBM Corp., Armonk, NY, USA). Descriptive statistics were employed to summarize baseline characteristics and clinical data. Survival curves were estimated using the Kaplan–Meier method and compared via log-rank test. Statistical significance was defined as a two-tailed *p*-value of less than 0.05.

## 3. Results

### 3.1. Baseline Characteristics

A total of 37 patients were included in the final analysis, and their baseline clinical characteristics are summarized in Table 1. The study population showed a male predominance, comprising 28 males (75.7%) and 9 females (24.3%). The median age at diagnosis was 62 years, with a wide range spanning from 12 to 88 years. Regarding age distribution, most patients were in the older group, with 26 patients (70.3%) aged 60 years or older. In contrast, younger populations were also represented: 2 patients (5.4%) were in their teens (10–19 years), 5 patients (13.5%) were between 20 and 49 years, and 4 patients (10.8%) were in their 50s.

### 3.2. Clinical Presentation

A substantial proportion of patients were asymptomatic at the time of diagnosis; 15 patients (40.5%) were referred due to incidental radiologic abnormalities, and 2 patients (5.4%) were identified through abnormal laboratory findings during a health check-up. Among symptomatic patients, abdominal discomfort was the most prevalent complaints (11 patients, 29.7%), followed by cholangitis (5 patients, 13.5%), and unintentional weight loss (4 patients, 10.8%) (Table 1).

Regarding serum pancreatic enzymes, the median levels of amylase and lipase were 85 U/L (range, 14–1290 U/L) and 162.5 U/L (range, 15–3551 U/L), respectively. Elevated amylase levels (≥100 U/L) were observed in 4 patients (10.8%), while elevated lipase levels (≥70 U/L) were identified in a higher proportion of 12 patients (32.4%). In terms of serum tumor markers, the median CEA level was 2.15 ng/mL (range, 0.5–5.3 ng/mL), with only 4 patients (10.8%) exceeding the threshold of 7 ng/mL. Notably, the median CA 19-9 level was 12.1 U/mL (range, 0.6–409 U/mL), most patients (71.9%) maintained levels within the normal reference range (Table 1).

### 3.3. Morphological and Radiological Features

**Figure 2:**
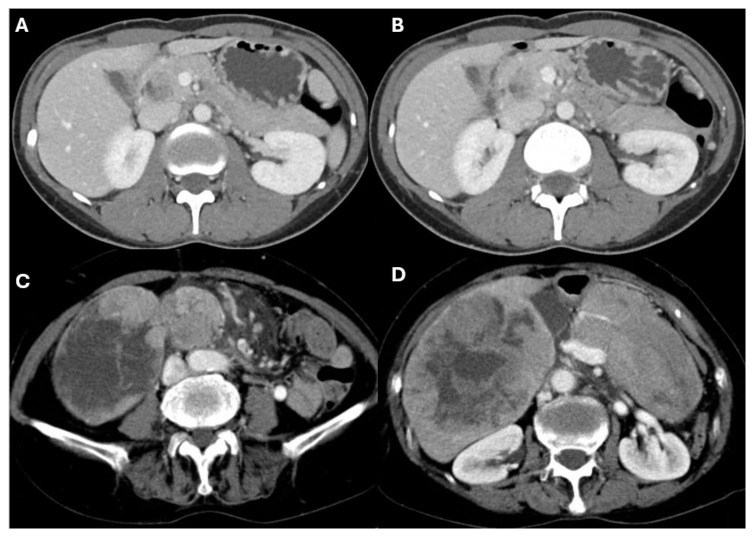
Computed tomography (CT) findings of pancreatic acinar cell carcinoma. (**A**,**B**) CT images in the arterial phases reveal a relatively well-defined, hypo-enhancing heterogenous solid mass in the pancreatic head/body without associated pancreatic duct dilation. (**C**) A large, heterogeneous mass is observed in the hepatic parenchyma, with internal low-density areas suggestive of central necrosis or cystic degeneration, accompanied by a huge mass in the pancreas head showing mixed enhancement features. (**D**) A mixed solid and cystic tumor replaces the right hepatic parenchyma, and a poorly defined tumor with necrotic components replaces a significant portion of the pancreatic body and tail.

**Figure 3:**
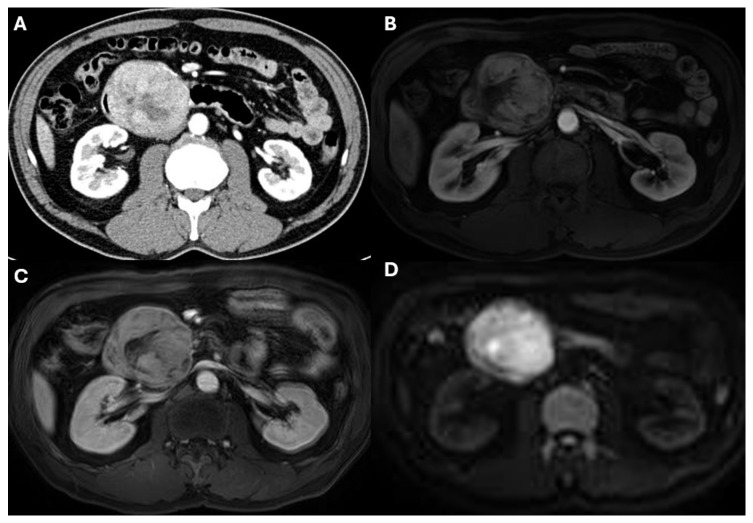
Radiologic assessment of a large pancreatic acinar cell carcinoma. (**A**) Axial arterial phase CT demonstrates a large, exophytic mass in the pancreatic head with prominent internal low-density areas, suggesting central necrosis. (**B**,**C**) Axial T1-weighted MR images obtained before and after Gadovist enhancement. The tumor shows a characteristic hypo-enhancing and heterogenous pattern. (**D**) Diffusion-weighted imaging reveals marked high signal intensity within the tumor.

The location of pancreatic ACC was evenly distributed throughout the pancreas: 13 (35.1%) were in the head, 13 (35.1%) in the body, and 11 (29.7%) in the tail. Most patients presented with a solitary mass (n = 31, 83.8%), while 5 patients (13.5%) had multiple lesions (Figure 2). The median tumor size was 47.3 mm (standard deviation [SD], 27.1 mm). Regarding the internal composition, most of the tumors exhibited a solid texture (n = 24, 64.9%), followed by mixed solid-cystic (10.8%) and purely cystic (10.8%) appearance. In terms of contrast enhancement, non-enhancing patterns were most prevalent, occurring in 18 patients (48.6%), while 13 patients (35.1%) showed heterogeneous enhancement (Figure 3). Internal calcification was found in only one patient (2.7%). Notably, pancreatic duct dilation was associated with the tumor in 17 patients (45.9%), a clinical feature requiring careful differentiation from PDAC. These radiologic features are summarized in Table 2.

### 3.4. Treatment Patterns According to Resectability

**Figure 4:**
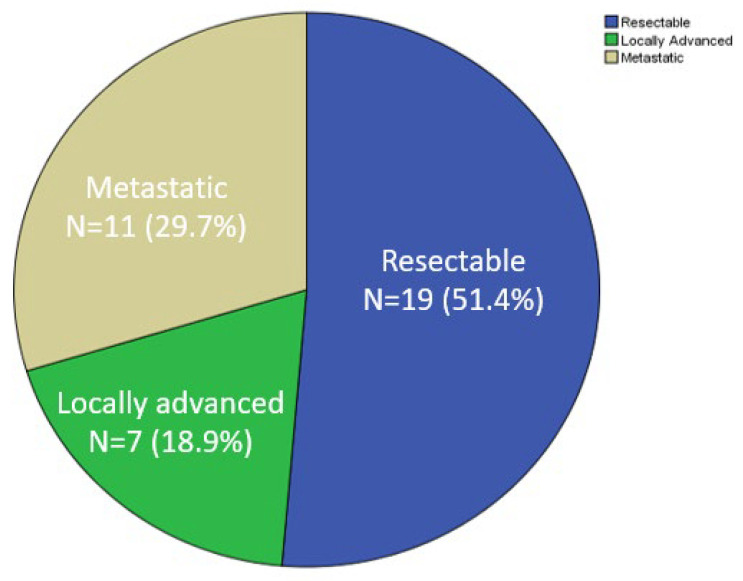
Distribution of clinical stages at the time of diagnosis. More than half of the patients presented with resectable disease (n = 19, 51.4%), followed by metastatic disease (n = 11, 29.7%) and locally advanced disease (n = 7, 18.9%).

At the time of diagnosis, patients were categorized based on disease resectability: 19 (51.4%) were classified as resectable, 7 (18.9%) as locally advanced, and 11 (29.7%) as metastatic (Figure 4). In the resectable group (n = 19), 17 patients (89.4%) underwent surgical resection, whereas one patient received palliative chemotherapy and another received best supportive care. Notably, in the locally advanced group (n = 7), four patients (57.1%) received surgical resection followed by adjuvant chemotherapy. The adjuvant regimens included gemcitabine monotherapy (n = 1), etoposide with cisplatin (n = 1), and gemcitabine plus nab-paclitaxel (n = 2). For the metastatic group (n = 11), systemic palliative chemotherapy was administered to 10 patients (90.9%). Among the patients with metastatic disease (n = 11), liver metastasis was the most common metastatic site and was present in 10 patients. Of these, 7 patients had both liver and lung metastases, whereas 1 patient had isolated lung metastasis. First-line chemotherapy regimens for locally advanced or metastatic stage were heterogeneous, including gemcitabine monotherapy, gemcitabine with nab-paclitaxel, FOLFIRINOX, etoposide with cisplatin, 5-FU with leucovorin. These treatment patterns are summarized in Table 3.

### 3.5. Survival Outcomes

**Figure 5:**
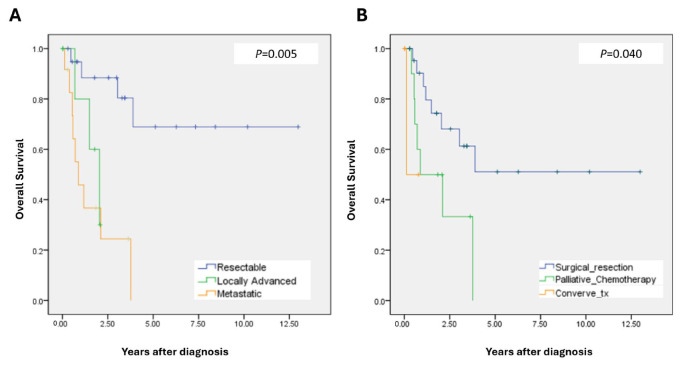
Kaplan–Meier survival curves for overall survival (OS). (**A**) OS stratified by initial clinical stage at diagnosis. Patients with resectable disease showed significantly superior survival outcomes compared to those with locally advanced or metastatic disease (Log-rank *p* = 0.005). (**B**) OS according to the primary treatment modality. Surgical resection was associated with a markedly better prognosis compared to palliative chemotherapy or best supportive care (Log-rank *p* = 0.040).

Survival analysis revealed a significant association with initial resectability (*p* = 0.005) (Figure 5A). For patients with resectable pancreatic ACC, the median overall survival (OS) was not reached during the follow-up period, and the 5-year OS rate was 60% (mean OS, 8.7 years). In contrast, patients with locally advanced or metastatic disease showed significantly shorter survival with median OS of 2.0 years and 0.9 years, respectively.

Regarding treatment strategy, surgical resection was performed in 22 patients (59.4%), including one case of palliative distal pancreatectomy for diagnostic confirmation. Palliative chemotherapy was administered to 12 patients (32.4%), while 4 patients (10.8%) received best supportive care. Survival analysis showed a significant difference in OS according to the treatment strategies (*p* = 0.040). For the patients who underwent surgical resection, the median OS was not reached, reflecting a remarkably prolonged survival (mean OS, 7.6 years; 5-year OS rate, 51%) (Figure 5B). In contrast, the palliative chemotherapy group had a median OS of 0.9 years, and the best supportive care group showed the most dismal prognosis with an OS of only 0.1 years.

Survival analysis according to resection margin status was attempted. However, no death events occurred in the resection margin positive group (n = 3) during follow-up; therefore, statistical comparison between the groups could not be reliably performed. However, KM survival analysis based on LN positivity showed that patients with N0 had not reached a median overall survival (OS), whereas those with N1 had a median OS of 37 months although the difference did not reach statistical significance.

### 3.6. Prognostic Factors for Overall Survival After Surgical Resection

Among the 22 patients who underwent surgical resection, including 1 Whipple procedure, 9 pylorus-preserving pancreatoduodenectomies, 11 distal pancreatectomies, and 1 mass enucleation, there was no significant prognostic factors for OS; lymphovacular invasion (Hazard Ratio [HR] 3.156, *p* = 0.139) and high FDG uptake on PET-CT (HR 105.972, *p* = 0.584) did not reach significance. However, pancreatic duct dilation and lymph node (LN) metastasis showed a trend toward poorer survival, with a HR of 3.728 (95% confidence interval [CI] 0.815–17.053; *p* = 0.090) and 4.369 (95% CI, 0.858–22.233; *p* = 0.076), respectively (Table 4). Pathologic results after surgical resection are summarized in Table 5.

## 4. Discussion

In the present study, the rarity of pancreatic ACC is underscored by the identification of only 37 cases across seven major academic referral hospitals over a 13-year period. This finding is consistent with a previous study from South Korea, which identified only 20 patients who underwent surgical resection for pancreatic ACC over 18-year period between 1997 and 2015 [6]. In contrast to PDAC, which is predominantly a disease of the elderly, our results demonstrated that pancreatic ACC exhibits a relatively broad age distribution, with 5.4% of patients in their second decade of life and 18.9% aged under 50 years. Furthermore, about 40% of enrolled patients were asymptomatic at the time of diagnosis. Morphologically, pancreatic ACC predominantly presented as solid, non-enhanced masses; specifically, 48.6% of tumors exhibited a non-enhancing pattern on CT. Notably, 45.9% showed associated ductal dilation, a feature that may complicate the differentiation from PDAC. However, atypical presentations were also observed, with 10.8% presenting as cystic masses and 13.5% manifesting as multiple lesions within the pancreas.

Regarding survival outcomes, the median OS for patients who underwent surgical resection for pancreatic ACC was not reached in this study, indicating a remarkably prolonged survival benefit. This finding is in line with the previous study, by Seo et al., which reported a median OS of 75 months (6.2 years). Our results further support the favorable long-term outcomes associated with curative-intent surgery for this rare malignancy [6]. Regarding prognostic factors, Seo et al. identified elevated CA 19-9 levels, lymph node metastasis, and perineural invasion as significant predictors of poor survival. The prognostic significance of LN involvement was further corroborated by a recently published multicenter study [7], in which LN involvement emerged as the sole statistically significant prognostic factor. In the present study, however, there were no statistically significant factors, although LN metastasis and pancreatic duct infiltration showed marginal significance. These differences may be attributed to the limited sample size inherent in studying such a rare disease, yet the observed trends align with existing evidence regarding the aggressive nature of pancreatic ACC with LN involvement.

The findings of our study provide several crucial take-home messages for clinical practice. Pancreatic ACC exhibits a broader age distribution, with approximately 20% of our cohort diagnosed under the age of 50. Radiologically, these tumors are characteristically present as large, hypo-enhancing solid masses on CT; however, a subset of pancreatic ACCs may exhibit an arterial hyperenhancement pattern or pleomorphic morphologies encompassing solid, cystic or mixed components. More importantly, despite their large size, pancreatic ACCs are relatively well-circumscribed and have a high resectability rate. Consequently, surgical resection affords a potential for long-term survival.

The substantial survival benefit derived from surgical resection has been constantly validated from several other studies [1,8,9,10]. In 2023, a multicenter European study involving 59 patients who underwent curative-intent resection reported that 5-year OS rate of 60.9% with the median OS not being reached during a median follow-up of 48 months [7]. Similarly, another multicenter retrospective study of Egal et al., which analyzed 44 patients with pancreatic ACC in France, demonstrated a median OS of 106.5 months (8.8 yrs) for those with non-metastatic state [11]. Furthermore, Wisnoski et al. analyzed 672 patients with pancreatic ACC from the Surveillance, Epidemiology, and End Results (SEER) database of the US, and reported a median OS of 123 months (10.25 years) for patients who underwent surgical resection [1]. However, it should be noted that these exceptionally favorable survival outcomes may be subject to bias, as the SEER has the potential for neuroendocrine tumors to be misclassified as ACC [1]. Taken together, the survival outcomes for pancreatic ACC treated with surgical resection appear to be more favorable than the historical data reported for PDAC. This marked survival advantage underscores the clinical importance of prioritizing surgical management whenever technically feasible.

Regarding clinical symptoms of pancreatic ACC, patients may present with lipase hypersecretion syndrome also known as “Schmid’s Triad.” This syndrome results from the massive systemic release of lipase from the pancreatic ACC, with serum levels often exceeding 10,000 U/L [2,12]. Furthermore, the systemic spillage of excessive pancreatic enzymes can cause autodigestion, leading to subcutaneous fat necrosis, which may be accompanied by peripheral blood eosinophilia, polyarthralgia or panniculitis [13,14]. However, pancreatic ACC more commonly presents with non-specific symptoms, such as abdominal discomfort, generalized weakness, and diarrhea [15]. Notably, painless jaundice or obstructive cholangitis—clinical hallmarks frequently observed in PDAC—are relatively uncommon in pancreatic ACC [8,16]. These observations were corroborated by the present study, where approximately half of the patients were asymptomatic at the time of diagnosis and were identified incidentally through abdominal imaging or laboratory findings. Furthermore, the low rate of CA 19-9 elevation observed in our study underscores the diagnostic challenges in identifying pancreatic ACC using conventional tumor markers.

Pancreatic ACC is characterized by well-circumscribed solid masses with poor contrast enhancements [17]. However, the present study demonstrated that pancreatic ACC can manifest with diverse morphological features, including cystic or mixed lesions, with or without associated pancreatic duct dilation. Furthermore, multifocal involvement within the pancreas may occur simultaneously. Previous literature has also reported atypical presentations, such as diffuse pancreatic enlargement resembling a “sausage-like” appearance [18], and rapid morphologic changes during the disease course due to internal tumor necrosis, even over short intervals [19]. Furthermore, a subset of pancreatic ACCs can occasionally exhibit an arterial hyperenhancement pattern—as observed in 8.1% of our cohort—which may cause them to radiologically mimic hypervascular pancreatic neuroendocrine tumors. These pleomorphic features have been constantly documented in several reports [10,17,18,19,20]. Given this morphologic diversity, relying solely on radiological features makes it challenging to distinguish pancreatic ACC from other pancreatic diseases, such as PDAC, neuroendocrine tumors or autoimmune pancreatitis.

Histologically, pancreatic ACC is reported to have low rates of LN metastasis, peri-neural invasion, and microvascular invasion. These findings can be attributed to the well-circumscribed histological structure, often characterized by fibrous septa, that sequester the tumor from the adjacent normal pancreas parenchyma. Consequently, these histologically features may reflect the less aggressive clinical behavior compared to that of PDAC [8]. In addition, approximately 40% of pancreatic ACC present with internal necrosis, which can also manifest during disease progression [19,21]. This phenomenon may be explained by the high density of tumor cells which require an adequate vascular supply to sustain their intense metabolic demands [15]. In contrast to conventional PDAC, which is characterized by an abundant fibrotic stroma and desmoplastic reaction, ACC typically has minimal stromal desmoplasia. Therefore, pancreatic ACC may be more directly influenced by alterations in tumor perfusion, potentially predisposing the lesion to rapid necrotic changes.

Moreover, pancreatic ACC may contain a neuroendocrine component. When neuroendocrine cells comprise more than 30% of the tumor, the malignancy is classified as “mixed acinar-neuroendocrine carcinoma” [10,22]. Consequently, it is noteworthy that immunohistochemical markers such as chromogranin A and synaptophysin immunohistochemistry can be detected in pancreatic ACCs. Although the expression of trypsin, chymotrypsin, and lipase was historically utilized for diagnostic confirmation, recent studies have reported that high expression of Bcl-10 (B-cell lymphoma/leukemia 10) provide superior diagnostic accuracy for identifying pancreatic ACC [10,23].

Currently, the optimal management of pancreatic ACC has not been firmly established due to its rarity; therefore, treatment strategies are often extrapolated from those used for PDAC. This pattern was also observed in the present study. However, pancreatic ACC has been reported to exhibit a lower frequency of *KRAS* and *TP53* mutations, which are predominant drivers in conventional PDAC [24,25]. Furthermore, a 2023 study by Mandelker et al. identified germline pathologic variants in homologous recombination/DNA damage repair genes in approximately 48% of patients with pure-type pancreatic ACC; notably, *BRCA2* variants were detected in 35% [26]. Therefore, homologous recombination deficiency may play a central role in the pathogenesis and progression of pancreatic ACC, providing a rationale for the use of platinum-based chemotherapy and PARP inhibitors in clinical practice.

This study has several limitations. First, the sample size was relatively small, which is an inherent constraint given the extreme rarity of pancreatic ACC. However, the sample size of 37 in this study is comparable to those reported in the previous literature [7,26,27]. For instance, a recent multicenter study conducted across 9 institutions in Europe included only 59 patients over two decades [7]. Second, the retrospective nature of this study may have introduced inherent selection bias. Nevertheless, to the best of our knowledge, this is the first multicenter study to include representative referral institutions in South Korea, thereby providing clinically significant insights into this rare malignancy. In addition, unlike prior studies that focused primarily on surgical cohorts, this study provides comprehensive evaluation of real-world treatment patterns, encompassing best supportive care and systemic therapy.

In conclusion, pancreatic ACC showed a broad age distribution—with approximately 20% of patients diagnosed at age under 50 years—and pleomorphic features, including solid, cystic, and mixed morphological patterns. Given its distinct biological profile and more favorable surgical outcomes compared with PDAC, pancreatic ACC should be recognized as a separate clinical entity requiring tailored management strategies. Based on the survival outcomes demonstrated in this study, surgical resection can offer significant potential to improve long-term survival, particularly for patients with resectable pancreatic ACC.

## Figures and Tables

**Table 1:** Baseline Characteristics and Clinical Features at Presentation.

Variables	
Male, n (%)	28 (75.7%)
Age at diagnosis, years (median, range)	62 (12–88)
Age distribution, n (%)	
- 10–19 years	2 (5.4%)
- 20–49 years	5 (13.5%)
- 50–59 years	4 (10.8%)
- 60 years or older	26 (70.3%)
Chief complaint, n (%)	
- Radiologic abnormality without symptoms	15 (40.5%)
- Abnormal laboratory findings without symptoms	2 (5.4%)
- Abdominal discomfort	11 (29.7%)
- Weight loss	4 (10.8%)
- Cholangitis	5 (13.5%)
Pancreatic enzymes and tumor markers	
- Amylase (U/L, median, range)	85 (14–1290)
- Amylase ≥ 100 U/L, n (%)	4 (10.8%)
- Lipase (U/L, median, range)	162.5 (15–3551)
- Lipase ≥ 70 U/L, n (%)	12 (32.4%)
- CEA ng/mL (median, range)	2.15 (0.5–5.3)
- CEA ≥ 5 ng/mL, n (%)	4 (10.8%)
- CA 19-9 U/mL (median, range)	12.1 (0.6–409)
- CA 19-9 ≥ 27 U/mL, n (%)	9 (28.1%)

**Table 2:** Morphological features of pancreas acinar cell carcinoma.

Variables	N = 37
- Location (head/body/tail), n (%)	13 (35.1%)/13 (35.1%)/11 (29.7%)
- Multiplicity (solitary/multiple), n (%) *	31 (83.8%)/5 (13.5%)
- Size, mm (median)	47.3 (27.1)
- Mass texture (solid/cystic/mixed), n (%) **	24 (64.9)/4 (10.8)/4 (10.8)
- Internal calcification, yes, n (%)	1 (2.7)
- Enhancement (Hyper/Hetero/Non-Enhanced), n (%) ***	3 (8.1)/13 (35.1)/18 (48.6)
- Pancreatic duct dilation, yes, n (%)	17 (45.9%)

Data were unavailable for 1 (*), 5 (**), and 3 (***) patients, respectively.

**Table 3:** Treatment patterns based on resectability of pancreas acinar cell carcinoma.

	Resectable (N = 19)	Locally Advanced (N = 7)	Metastatic (N = 11)
Surgical resection	17 (89.4%)	4 (57.1%) *	1 ** (8.3%)
Palliative CTx	1 (5.2%)	1 (14.3%)	10 ** (83.3%)
Best supportive care	1 (5.2%)	2 (28.6%)	1 (8.3%)

* Adjuvant chemotherapy with gemcitabine monotherapy (n = 1), etoposide with cisplatin (n = 1), gemcitabine with nab-paclitaxel (n = 2). ** One patient with distal pancreatectomy for diagnostic confirmation and treated with palliative chemotherapy.

**Table 4:** Prognostic factors for overall survival after surgical resection.

	HR	95% CI	*p*-Value
Morphological features			
Pancreatic duct dilation (+)	3.728	0.815–17.053	0.090
Mixed pattern (+)	1.198	0.106–13.549	0.884
Multiplicity (+)	2.432	0.571–10.355	0.229
Pathologic results			
Resection margin (+)	0.040	0.001–629.174	0.514
Lymphovascular invasion	3.156	0.689–14.452	0.139
Lymph node (+)	4.369	0.858–22.233	0.076
Initial laboratory findings			
CEA (+)	1.974	0.205–19.046	0.557
CA 19-9 (+)	0.485	0.057–4.164	0.510
Amylase/lipase (+)	1.977	0.216–018.060	0.546
PET-high uptake	105.972	0.000–1909 × 10^6^	0.584
Adj CTx (+)	0.296	0.030–2.871	0.294

**Table 5:** Pathologic results after surgical resection.

Pathological Variables	Total (n = 19) *	Tumor Size ≤ 2 cm (n = 1)	Tumor Size 2–4 cm (n = 9)	Tumor Size > 4 cm (n = 9)
Lymph node metastasis (N+)	6/19 (31.6%)	0/1 (0.0%)	4/9 (44.4%)	2/9 (22.2%)
Lymphovascular invasion (+)	6/19 (31.6%)	0/1 (0.0%)	4/9 (44.4%)	2/9 (22.2%)
Perineural invasion (+)	5/19 (26.3%)	0/1 (0.0%)	3/9 (33.3%)	2/9 (22.2%)
Positive resection margin (R1)	3/19 (15.7%)	0/1 (0%)	1/9 (11.1%)	2/9 (22.0%)

* Pathological data were unavailable for three patients.

## Data Availability

Data available on request due to restrictions. The data presented in this study are available on reasonable request from the corresponding author due to ethical restrictions and patient privacy concerns.
